# Enhancing Local Functional Structure Features to Improve Drug–Target Interaction Prediction

**DOI:** 10.3390/ijms262010194

**Published:** 2025-10-20

**Authors:** Baoming Feng, Haofan Du, Henry H. Y. Tong, Xu Wang, Kefeng Li

**Affiliations:** 1 Center for Artificial Intelligence Driven Drug Discovery, Faculty of Applied Sciences, Macao Polytechnic University, Macao 999708, China; p2521368@mpu.edu.mo (B.F.); henrytong@mpu.edu.mo (H.H.Y.T.); 2 School of Physics and Technology, Nanjing Normal University, Nanjing 210023, China; 07220318@njnu.edu.cn; 3 State Key Laboratory of Food Nutrition and Safety, College of Food Science and Engineering, Tianjin University of Science and Technology, Tianjin 300457, China

**Keywords:** drug–target interaction, molecular simulation, local functional structures, gated cross-attention, neural network, attention mechanism

## Abstract

Molecular simulation is central to modern drug discovery but is often limited by high computational cost and the complexity of molecular interactions. Deep-learning drug–target interaction (DTI) prediction can accelerate screening; however, many models underuse the local functional structure features—binding motifs, reactive groups, and residue-level fragments—that drive recognition. We present LoF-DTI, a framework that explicitly represents and couples such local features. Drugs are converted from SMILES into molecular graphs and targets from sequences into feature representations. On the drug side, a Jumping Knowledge (JK) enhanced Graph Isomorphism Network (GIN) extracts atom- and neighborhood-level patterns; on the target side, residual CNN blocks with progressively enlarged receptive fields, augmented by N-mer substructural statistics, capture multi-scale local motifs. A Gated Cross-Attention (GCA) module then performs atom-to-residue interaction learning, highlighting decisive local pairs and providing token-level interpretability through attention scores. By prioritizing locality during both encoding and interaction, LoF-DTI delivers competitive results across multiple benchmarks and improves early retrieval relevant to virtual screening. Case analyses show that the model recovers known functional binding sites, suggesting strong potential to provide mechanism-aware guidance for molecular simulation and to streamline the drug design pipeline.

## 1. Introduction

Since its inception in the 1950s with foundational techniques like Monte Carlo sampling and molecular dynamics, molecular simulation technology (MST) has become an indispensable tool across scientific disciplines, including pharmacology, phytomedicine, and molecular physics [1,2,3,4]. In the realm of health promotion, MST is particularly valuable for exploring the interaction mechanisms between bioactive compounds and therapeutic protein targets, as well as interactions between bioactive compounds and their environments [5]. By providing atomic-level insights, it significantly reduces the labor and resource costs associated with traditional experimentation. However, the utility of MST for large-scale applications, such as screening vast compound libraries, remains hampered by substantial computational burdens, creating a critical bottleneck in the discovery pipeline [6].

In response to these computational demands, the rapidly evolving field of machine learning (ML) offers powerful approximation methods for complex atomic systems, effectively bypassing the need to solve computationally intractable equations [7]. First, traditional structure-based approaches, most notably molecular docking with physics/empirical/knowledge-based scoring functions explicitly model the 3D binding pose and approximate binding energies when reliable protein structures are available; these methods provide physical interpretability but depend on structural availability/quality and can be sensitive to receptor flexibility and scoring errors [8]. Second, data-driven learning approaches infer interactions from observed patterns. Among them, classic kernel-based baselines offer data efficiency when informative similarity matrices are available, albeit with reliance on handcrafted similarities and potential cold-start limitations [9]. While deep learning (DL)-based DTI techniques leverage known interaction data and molecular features to learn rich representations and rapidly estimate binding affinity between compounds and targets [10]. By providing an efficient means for preliminary screening and prioritization, these DTI models guide subsequent in-depth molecular simulation studies and experimental validation [11].

Recent advancements in DL have led to a suite of sophisticated DTI models that learn from heterogeneous data sources like chemical structures and protein sequences [12]. MolTrans [13] uses knowledge-guided substructure mining and an enhanced Transformer to model interactions. MCANet [14] adopts a cross-attention mechanism to enhance drug–target feature associations. DrugBAN [15] introduces a bilinear attention network with domain-adaptive learning to improve generalization. DefuseDTI [16] employs a dual-branch encoder and multi-view attention to extract fine-grained multimodal features. DMHGNN [17] builds a dual-view heterogeneous GNN to learn drug–protein representations via topological and semantic graphs. MHAN-DTA [18] proposes a multiscale hybrid attention network to enhance feature extraction capabilities, thereby improving the performance of affinity prediction. Multi-ITI [19] integrates a pre-trained biological feature learning module with a heterogeneous graph neural network equipped with dynamic graph attention, effectively capturing drug–target network relationships while mitigating the impact of noise.

However, despite their impressive performance, a critical limitation persists: most existing models do not explicitly enhance the representation of local functional structures of compounds or targets. They often focus on global representations or abstract interaction patterns, thereby overlooking the fine-grained, functionally relevant substructures, such as reactive groups, binding motifs, or local sequence/graph neighborhoods—that ultimately govern molecular recognition and binding [20]. The importance of focusing on local features is gaining attention. For example, Liu et al. [21] employed recurrent neural networks to capture intrinsic features and dependencies within sequences, and introduced incremental learning to improve the adaptability and accuracy of miRNA prediction. Gao et al. [22] incorporated pathway information and contrastive learning [23] into spatial transcriptomics studies, taking into account the local functional correlations between spatial points to better uncover underlying biological characteristics.

To address this gap and better support MST-driven discovery, we propose LoF-DTI, a deep learning model explicitly architected to strengthen local functional structure features. The model systematically decomposes inputs on both sides: drug SMILES are converted to molecular graphs and encoded by a Graph Isomorphism Network (GIN) with Jumping Knowledge to capture hierarchical, atom- and neighborhood-level patterns in drugs [24], while protein sequences are processed by a CNN-based residual [25] module. We further augment locality with N-mer substructural semantic features, which emphasize motif-scale signals in both molecules and sequences. The resulting drug and target representations are fused by a Gated Cross-Attention (GCA) module that uses multi-head attention to highlight interaction cues between key atoms and residues, and a gating mechanism to adaptively balance raw local evidence with global context. This design improves the identification of pharmacophores and yields high-confidence, interpretable structure–activity associations to guide downstream molecular simulation and experimental validation. In contrast to approaches that rely primarily on global similarity or coarse feature aggregation, our method explicitly prioritizes local functional structures during both representation learning and cross-modal interaction, enabling precise alignment of atom–residue signals. This design not only improves the identification of key pharmacophores but also provides high-confidence, interpretable structure-activity associations to guide subsequent molecular simulation experiments.

## 2. Results and Discussion

### 2.1. Performance Evaluation

To benchmark the predictive power of LoF-DTI, we conducted a comparative analysis against six state-of-the art baseline models—DeepConv-DTI [26], GraphDTA [27], MolTrans [13], DrugBAN [15], NFSA-DTI [28], and IHDFN-DTI [29] across four benchmark datasets. The experimental results are presented in Table 1, where the bolded **values** indicate the best performance and underlined values denote the second-best results.

As shown in Table 1, LoF-DTI consistently achieves the best overall performance across all four benchmark datasets, demonstrating its superior ability to model complex drug–target interactions. Across the BindingDB, BioSNAP, DAVIS, and Human datasets, LoF-DTI achieves the highest scores in most evaluation metrics, including AUROC, AUPRC, and Accuracy. Among them, the low AUPRC on the DAVIS dataset is primarily driven by evaluation and distributional factors that are specific to DAVIS, a low positive prevalence combined with a thresholding strategy that makes AUPRC highly sensitive. Notably, while IHDFN-DTI shows competitive results on some metrics, LoF-DTI achieves its state-of-the-art performance with a model that is four times smaller in parameter size. This underscores the exceptional computational efficiency of our proposed architecture. Collectively, these findings establish LoF-DTI as a robust, generalizable, and highly efficient framework that strikes an optimal balance between predictive accuracy and model complexity.

### 2.2. Ablation Studies

To dissect the contribution of each architectural component to the overall performance of LoF-DTI, we conducted a comprehensive series of ablation studies. The study systematically evaluated the impact of removing key components: the Jumping Knowledge (JK) mechanism, the residual connections (RS) in the protein encoder, and the entire Gated Cross-Attention (GCA) module. We also replaced the Graph Isomorphism Network (GIN) with a standard Graph Convolutional Network (GCN) to assess its specific contribution.

**Figure 1:**
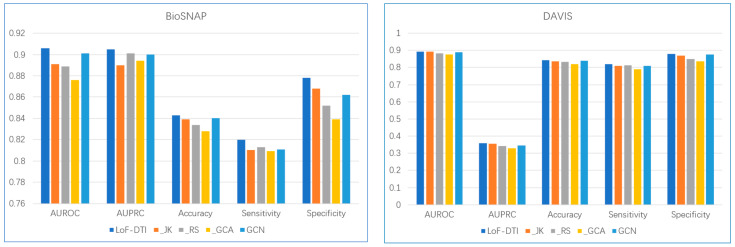
Ablation study on different modules.

Figure 1 presents the results of ablation studies on different modules. In the figure, “_JK” denotes the removal of the JK mechanism from the full model; “_RS” indicates the removal of residual connections, replacing the structure with a basic CNN; “_GCA” represents the absence of the GCA module, where drug and target features are directly concatenated instead; and “GCN” refers to replacing GIN with a standard GCN [30]. During each ablation experiment, all other components remain consistent with the original LoF-DTI configuration. All experimental results presented in Figure 1 were obtained using consistent hyperparameter configurations.

Every tested component contributes positively to the model’s performance. The most substantial performance drop was observed upon removal of the GCA module, highlighting its critical role in learning effective drug–target interaction representations. Compared to simple concatenation or averaging strategies, GCA effectively highlights functional interaction regions between drugs and targets, thereby greatly enhancing performance in both classification and regression tasks. Furthermore, the DrugGIN and ProteinRS modules provide strong unimodal representations that serve as high-quality inputs to the GCA module, demonstrating the synergy between the specialized encoders and the interaction module.

**Figure 2:**
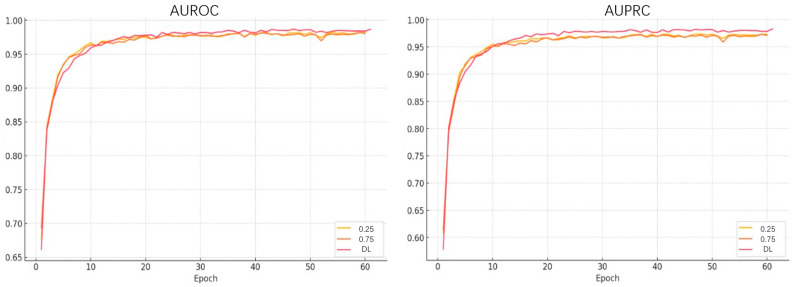
Impact of gating strategies on model performance.

Given the importance of the GCA module, we further investigated the impact of different gating strategies on the Human dataset. Figure 2 shows the AUROC and AUPRC values on the validation set over the first 60 training epochs.

The results demonstrate that the dynamic gating mechanism consistently outperforms other gating strategies, maintaining higher AUROC and AUPRC scores throughout the training process. It also exhibits better stability and faster convergence. This suggests that incorporating learnable dynamic weights during feature fusion enables the model to adaptively adjust the importance of drug and target information for each specific sample. Such adaptability helps the model capture critical interaction signals more accurately. Compared to static or averaged gating approaches, the dynamic mechanism more effectively distinguishes between redundant and informative features, leading to improved overall prediction performance. These findings further confirm the pivotal role of the GCA module in enhancing the model’s discriminative capability.

**Figure 3:**
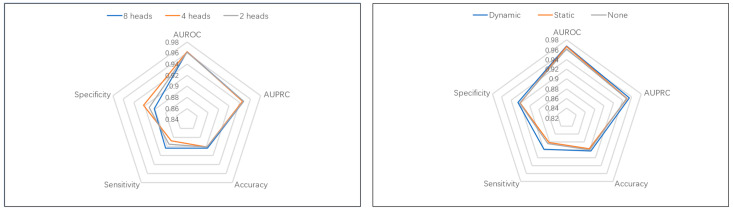
Impact of N-mer features and the number of attention heads in the GCA module on model performance.

Further ablation studies, shown in Figure 3, explored the sensitivity of LoF-DTI to hyperparameters and the impact of N-mer features. While the number of attention heads had a modest effect on performance, the inclusion of 3-mer substructural features provided a consistent and significant boost across all metrics, particularly in sensitivity and accuracy. Notably, dynamically learned projections for these features were essential, as static random mappings offered minimal benefit, confirming the value of adaptive feature engineering in our model.

### 2.3. Case Studies

#### 2.3.1. DTI Prediction as a Guide for Molecular Simulation

Traditional techniques such as molecular simulation are often limited in their ability to efficiently screen potential compounds in advance. In contrast, our model leverages large-scale learning of relevant knowledge to effectively identify candidate compounds that are likely to interact with specific drug molecules or protein targets, thereby avoiding repetitive and aimless experimental efforts [31].

We conducted a case study involving the drug Sertraline and the complex 3QMN. Sertraline is a selective serotonin reuptake inhibitor, primarily used for the treatment of depression, anxiety disorders, obsessive–compulsive disorder, and related mood disorders [32]. The complex 3QMN corresponds to the crystal structure of 4′-phosphopantetheinyl transferase AcpS from Vibrio cholerae O1 biovar eltor [33].

In Table 2, the model produces affinity scores above 0.9 for all drug–target and target–compound pairs, indicating predicted matches. Specifically, the interaction between Sertraline and its corresponding target is validated by DrugBank [34], while the interaction between the target 3QMN and its associated compound is verified using the PDB [35]. Given that our model is capable of learning from large-scale data, it can serve as an efficient and accurate initial guide for MS, such as predicting potential drug–target binding modes or screening high-confidence candidate molecules. This significantly reduces the computational resources and time costs required by traditional simulation techniques. In comparison to conventional approaches, our method offers data-driven prior knowledge, enabling dual improvements in both simulation accuracy and efficiency [36].

#### 2.3.2. Interpretable Prediction of Functional Structures

Beyond predicting interaction likelihood, a central goal of LoF-DTI is to provide interpretable, atom-level insights to directly guide molecular simulations. To more effectively support traditional methods such as MS, LoF-DTI provides targeted guidance at the molecular level. We extracted the “Att map” from the GCA module, which captures the correlation between target residues and individual atoms within the drug molecule. After removing virtual nodes, we traversed the attention matrix and selected the top 20% of high-weight indices. These indices were then mapped back onto the molecular graph of the drug to identify the most critical atomic regions involved in the specific drug–target interaction. This process provides precise structural insights that can guide downstream tasks in molecular simulation, such as docking and conformational sampling.

**Figure 4:**
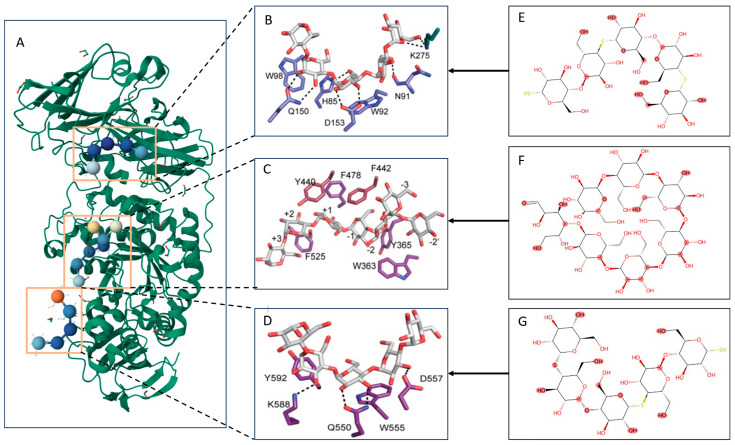
LoF-DTI Predicted Key Atom Visualizations and Validation Images. (**A**) Overview of the 8DL1 complex, with Maltoheptaose in the binding domain shown as colored spheres. (**B**–**D**) Validated binding site visualizations. (**E**–**G**) Key functional structures predicted by LoF-DTI, highlighted in red.

As shown in Figure 4, we conducted a case study on the8DL1 complex (BoGH13ASus-E523Q from *Bacteroides ovatus* bound to maltoheptaose) to validate the interpretability and practical applicability of our model predictions.

The upper and lower ends of the complex are composed of Maltopentaose, while the central ligand is Maltoheptaose. Although the structures may appear complex, they are essentially oligosaccharide-based organic compounds. Oligosaccharides, consisting of 3 to 10 monosaccharide units linked by glycosidic bonds, have a relatively simple elemental composition, allowing focused analysis of functional moieties with minimal interference from rare elements [42]. For the prediction involving Maltopentaose, we input only the protein sequences from the upper and lower domains into the model. In contrast, for Maltoheptaose, the complete protein sequence was used as input.

According to relevant biochemical experiments and literature [43], the classical catalytic pocket is composed of a catalytic triad—D477, E523 (mutation site E523Q), and D581—surrounded by aromatic residues (Y365, F478, Y440, F442, F525) that precisely position the oligosaccharide substrate at the −1 to +2 subsites through π–π interactions and hydrogen bonds. Additionally, an α-1,6-branched glucose at the −2 position (−2′) is specifically recognized and stabilized via a hydrogen bond network involving R641, the backbone of W363, and the side chain of N366. Two newly identified sites include: (1) an aromatic platform on the N-terminal CBM98 module (residues 44–163), formed by W92 and W98, which interacts with the O2/O3 atoms of the oligosaccharide through hydrogen bonding with polar residues; and (2) a surface site on the catalytic domain, located at W555 and Y592, which binds to a pentasaccharide unit via direct hydrogen bonding and water-mediated bridges.

LoF-DTI successfully predicted all these critical sites. The extracted attention weights were significantly enriched around the catalytic pocket and the newly identified sites, indicating that the model not only captures known functional residues but also learns potential structure–function association patterns from complex 3D structures. These results demonstrate the potential of LoF-DTI to assist in experimental structural-function annotation and the identification of novel active sites, providing strong support for a deeper understanding of drug–target mechanisms of action.

## 3. Materials and Methods

### 3.1. Evaluation Metrics and Implementation

The performance of LoF-DTI was evaluated using five metrics: Area Under the Receiver Operating Characteristic Curve (AUROC), Area Under the Precision–Recall Curve (AUPRC), accuracy, sensitivity, and specificity. Among them, AUROC (Area Under the Receiver Operating Characteristic Curve) and AUPRC (Area Under the Precision-Recall Curve) serve as the primary indicators of classification effectiveness. AUROC reflects the trade-off between true positive and false positive rates across varying thresholds, while AUPRC highlights the balance between precision and recall. Additionally, accuracy, sensitivity, and specificity are reported at the threshold corresponding to the optimal F1 score. The definitions of metrics are as follows:

(1) $$Sensitivity=\frac{FP}{FP+TN},$$

(2) $$Specificity=\frac{TP}{TP+FN},$$

(3) $$Precision=\frac{TP}{TP+FP},$$

(4) $$Accuracy=\frac{TP+TN}{TP+FP+TN+FN},$$

here, TP, TN, FP,FN denote true positives (The model predicts positive and the pair is labeled positive in the dataset/by the thresholding rule), true negatives (The model predicts negative and the pair is labeled negative), false positives (The model predicts positive but the pair is labeled negative, either measured inactive or below the activity threshold), and false negatives (The model predicts negative but the pair is labeled positive), respectively. In general, higher values across these metrics indicate better model performance. This work focuses on prediction correctness rather than structural geometric accuracy; therefore, we do not employ interface-level structural metrics such as I-RMSD [44] or I-INF [45]. These metrics require ground-truth three-dimensional complex structures, which are not available under the current data conditions.

Our experimental setup was deployed on a system equipped with two RTX 4070 units, made by NVIDIA in the USA, each with specifications of 8 GB of graphics memory, 12 virtual CPUs, and 32 GB of RAM. The execution environment and versions of tools adopted by LoF-DTI include: PyTorch 2.3.2+CUDA 11.8, Python 3.9.23, DGL 0.9.1post1, Numpy 1.23.5, Pandas 1.5.3, and RDKit 2022.09.5. Key hyperparameter settings are summarized in Table 3.

### 3.2. Datasets

We evaluated the performance of LoF-DTI and six state-of-the-art baseline models on four benchmark datasets: BindingDB [46], BioSNAP [47], Human [48], and DAVIS [49]. Detailed statistics for each dataset are provided in Table 4.

Each dataset was randomly split into training, validation, and test sets with a fixed ratio of 7:1:2. The test sets consist exclusively of unseen drug–target pairs. For each dataset, we conducted five independent experiments. The model achieving the best AUROC on the validation set was selected and subsequently evaluated on the test set to report the final performance.

### 3.3. Method

**Figure 5:**
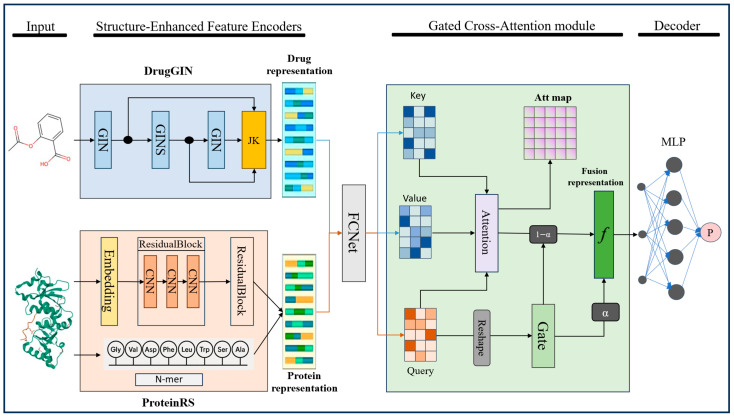
The Architecture of the LoF-DTI Model. Drug SMILES strings are converted into molecular graphs, while protein sequences are mapped into feature matrices. The DrugGIN module employs a multi-layer GIN combined with a Jumping Knowledge mechanism to effectively extract local structural features from drug molecular graphs. The ProteinRS module leverages a residual convolutional network to extract protein representations that preserve local domain-level information from the amino acid sequence, while concurrently incorporating N-mer-level substructural semantic features to capture scale patterns. Subsequently, the GCA module facilitates cross-modal feature interaction between drugs and targets, highlighting the coupling relationships of key functional regions. The fused features f are finally passed through a MLP classifier to predict potential drug–target interactions.

LoF-DTI comprises two main modules, as shown in Figure 5. First, DrugGIN and ProteinRS encode the drug and the target, respectively, extracting features that capture fine-grained, functionally relevant substructures (e.g., molecular motifs and active-site residues). Second, the GCA module further abstracts and fuses these features, strengthening the representation, interaction modeling, and alignment of local functional structures between small molecules and their protein targets. This design not only improves DTI predictive accuracy but also enhances structural-level interpretability: the GCA module’s attention mechanism computes atom-to-residue relevance for every drug–protein pair, enabling us to attribute binding interactions to specific regions of the compound and of the protein.

#### 3.3.1. Structure-Enhanced Drug Feature Encoder

To effectively capture the local structural characteristics of drug molecules, we propose a structure-enhanced feature encoder based on the GIN. This encoder first translates each atom’s chemical properties into a numerical format that a neural network can process, and then uses the GIN architecture to learn from the molecules’ graph structure.

Step 1: From SMILES String to Raw Atomic Features

The process begins with the drug’s SMILES string, a standard text-based representation of a molecule. Using the RDKit library, we parse this string to create a computational molecular graph, where atoms are nodes and bonds are edges. For each atom (node) in this graph, we extract a set of 8 key chemical attributes: its atom type (e.g., Carbon, Oxygen), atomic degree (number of bonds), number of implicit hydrogens, formal charge, number of radical electrons, hybridization state (e.g., sp^2^, sp^3^), total number of attached hydrogens, and an indicator for whether it is part of an aromatic ring. These attributes are then converted into a single numerical vector for each atom. For instance, categorical features like atom type are one-hot encoded, while others are represented as integers. By concatenating all these numerical values, we create a 74-dimensional feature vector for every atom in the molecule. For a drug molecule containing N atoms, we stack these individual vectors to form an input feature matrix, which we denote as X. The dimensions of this matrix are N × 74.

Step 2: Learning Initial Atom Embeddings via Linear Projection

This raw feature matrix X is sparse and high-dimensional. It represents chemical properties in a way that is human-readable but not optimized for a neural network. For example, the raw numbers do not inherently capture the chemical similarity between different elements. Therefore, our first task is to transform this raw data into a more compact, dense, and meaningful representation—a process known as creating an “embedding.”

We achieve this using a simple linear transformation followed by a non-linear activation function (ReLu), as shown in Equation 5. This is a standard input layer in a neural network.

(5) $$H^{\left(0\right)}=ReLu(XW_{0}),$$

X is the input feature matrix described above, with dimensions (N, 74), where N is the number of atoms. $W_{0}$ is a learnable weight matrix with dimensions. The values in this matrix are not fixed but are automatically adjusted during model training. The network learns the optimal transformation to convert the 74 raw features into meaningful features. The matrix multiplication $XW_{0}$ projects each atom’s 74-dimensional raw feature vector into a new dimensional vector. ReLU is the Rectified Linear Unit activation function, a standard component that introduces non-linearity, allowing the model to learn more complex patterns. $H^{\left(0\right)}$ is the resulting output matrix, with dimensions. Each row of $H^{\left(0\right)}$ is now a dense vector, or “embedding,” that represents an atom in a way that is optimized for the subsequent neural network layers. This matrix serves as the initial node representation for the drug molecule. For processing molecules in batches, all molecular graphs are padded with zero-valued dummy nodes to a fixed maximum node count.

Step 3: Aggregating Neighborhood Information with GIN and Jumping Knowledge

With these initial atom embeddings $H^{\left(0\right)}$, we then use a stack of Graph Isomorphism Network (GIN) layers to extract structure-aware representations. Each GIN layer updates an atom’s feature vector by aggregating information from its immediate neighbors

(6) $$h_{v}^{\left(k\right)}=\mathrm{ML}P^{\left(k\right)}\left((1+\varepsilon^{\left(k\right)})\cdot h_{v}^{\left(k-1\right)}+\sum_{u\in N(v)}h_{u}^{\left(k-1\right)}\right),$$

where $h_{v}^{\left(k\right)}\;$ is the feature vector of node v at layer k, N(v) denotes the neighbors of node v, MLP is a multi-layer perceptron, and ε is a learnable parameter. After each GIN layer, batch normalization and ReLU activation are applied to stabilize training.

To alleviate the limitations of deep GNNs in capturing hierarchical features and to enhance gradient flow, we incorporate JK aggregation. Specifically, the outputs of all GIN layers are concatenated and projected back to a unified feature space:

(7) $$h_{JK}=W_{JK}\cdot Concat\left(h^{\left(1\right)},h^{\left(2\right)},\ldots ,h^{\left(K\right)}\right),$$

$W_{JK}$ denotes the weight matrix for the linear transformation applied after JK aggregation. This allows the model to flexibly combine information from different receptive field sizes and network depths, enriching the encoded representation. These node-level embeddings are then passed to downstream modules for further fusion or prediction tasks.

#### 3.3.2. Structure-Enhanced Protein Feature Encoder

To extract informative and structure-aware representations from protein sequences, we design a convolutional encoder named ProteinRS, which leverages residual convolutional blocks to enhance the hierarchical modeling of local sequential patterns. This encoder not only captures local motifs in amino acid sequences but also preserves gradient flow and alleviates degradation through residual connections. This structure-aware design makes it particularly effective in modeling the biochemical and functional relevance of amino acid sequences in tasks such as drug–target interaction prediction.

Each protein sequence is treated as a string over a 25-character alphabet representing amino acids. Let $D_{p}$ denote the dimensionality of the target (protein) latent space. We initialize a learnable embedding matrix $X_{V}\in R^{25\times D_{p}}$ covering all 25 amino-acid tokens. By looking up $X_{V}$, each target is encoded into an initial feature matrix. Analogous to the drug side, we then apply a simple linear projection to obtain the dense target input features $X_{p}$:

(8) $$X_{p}=\sigma (W_{0}X_{d}),\;X_{p}\in R^{\Theta_{p}\times D_{p}}$$

Each residual block is composed of a sequence of 1D convolutional layers with batch normalization, activation, and dropout, the residual block computes:

(9) $$Y=\sigma \left(F(X)+G(X)\right),$$

where F(⋅) denotes the stacked convolutional transformation, G(⋅) denotes a dimension-matching shortcut connection. After the residual blocks, the feature maps are transposed and reshaped to fit downstream processing requirements. This residual design ensures both feature richness and gradient stability during training.

To effectively extract local functional structure information from protein sequences, we adopt a 3-mer-based statistical encoding scheme combined with a random projection-based dimensionality reduction method. This approach preserves local sequence patterns while projecting high-dimensional representations into a more compact embedding space, enhancing efficiency and maintaining functional relevance.

Given a protein sequence composed of characters from an alphabet of 25 amino acid symbols, we first enumerate all possible combinations of 3 continuous characters. The total number of possible 3-mer patterns is $D=25^{3}=15,625$. Each input sequence is then scanned with a sliding window of size 3 to extract all its 3-mers. We define a count vector $x\in {\mathbb{R}}^{D}$ for each sequence, where each element $x_{i}$ corresponds to the frequency of the i-th 3-mer in the sequence:

(10) $$x_{i}=\frac{n_{i}}{\sum_{j=1}^{D}n_{j}},$$

where $n_{i}$ is the number of times the i-th 3-mer appears in the sequence. This normalization ensures that $\sum_{i=1}^{D}x_{i}=1$, making the representation invariant to sequence length.

To mitigate the curse of dimensionality and reduce computation cost while retaining discriminative power, we employ a random projection matrix. Each element of R is sampled from a Gaussian distribution:

(11) $$R_{ij}\sim N\left(0,\frac{1}{\sqrt{d}}\right).$$

This operation projects the original high-dimensional k-mer frequency vector into a lower-dimensional space while approximately preserving pairwise distances and inner products. Unlike fixed handcrafted descriptors or learned embeddings that may overlook local motifs, our method explicitly models short-range interactions via 3-mers, aligning well with the notion of local functional domains in proteins.

#### 3.3.3. Gated Cross-Attention Module

To enable fine-grained and structure-aware interactions between drug and protein representations, we propose a GCA module. This module performs multi-head attention from one modality (query) to another (key-value), followed by a learnable fusion mechanism that selectively combines the attended features with the original query. This design allows for dynamic and adaptive integration of cross-modal information while preserving the semantics of the original features.

Set a query $Q=X_{p}$, a key $K=X_{d}$, and a value $V=X_{d}$, these representations are then reshaped for multi-head attention [50], where hidden dimension is $D_{H}$.

(12) $$Q^{\prime }=W_{Q}Q,\;K^{\prime }=W_{K}K,\;V^{\prime }=W_{V}V,$$

here, $W_{Q}\in R^{L_{q}\times D_{H}}$, $W_{K}\in R^{L_{k}\times D_{H}}$, $W_{V}\in R^{L_{v}\times D_{H}}$. For each $head_{i}$, the attention weights are computed as:

(13) $$Attention(Q^{\prime },K^{\prime },V^{\prime })=Soft\max\left(\frac{Q^{\prime }{K^{\prime }}^{⊤}}{\sqrt{d_{h}/h}}\right)V^{\prime }.$$

After applying attention across all heads, the output is concatenated and linearly transformed:

(14) $$A=Linear(Concat(head_{1}\;,\ldots ,head_{h}\;)).$$

To integrate the attended features A with the original query Q, we introduce gated fusion strategy. This strategy enables token-level, context-aware control over how much attention-driven interaction should influence the final representation, allowing the model to adaptively balance between self-information and cross-modal context:

(15) H=α⋅A+(1−α)⋅Proj(Q),α∈(0,1)

here, Proj(·) refers to the linear mapping function used to transform feature dimensions. α can be manually fine-tuned according to the task type, or it can be learned adaptively by the model. The formulation for dynamic adaptive learning is as follows:

(16) α=Sigmoid(FcNet([A;Proj(Q)])).

The GCA Module is a core component in our framework for drug–target interaction modeling. This design enables the model to learn both shared and complementary patterns from cross-modal pairs, significantly improving downstream prediction performance.

## 4. Conclusions

In this study, we introduced Local Functional Structure-aware Drug–Target Interaction, a deep-learning framework that strengthens both the modeling and the alignment of local functional structure features for drug–target interaction prediction. On the drug side, a Jumping-Knowledge-enhanced Graph Isomorphism Network extracts atom-level and neighborhood-level patterns from molecular graphs. On the target side, a convolutional neural network-based residual encoder captures multi-scale sequence motifs, further reinforced with N-mer substructural semantics to emphasize locality. A gated cross-attention module then performs bidirectional, multi-head cross-attention between atom and residue tokens; for each side it forms a cross-attended summary from the other side and uses a learnable gate to modulate the contribution of this cross-attended signal relative to the original local embedding, after which the gated summaries are pooled to obtain a fused representation with token-level attribution. Across four benchmark datasets, the method consistently surpasses six comparative approaches, achieving the best overall performance while maintaining a compact model size. Case studies show that it accurately identifies known catalytic residues and suggests previously unreported functional sites, offering mechanism-aware hypotheses with high confidence. Overall, prioritizing local functional structure features improves both accuracy and interpretability and provides precise guidance for downstream molecular simulation and experimental design, helping to streamline the drug discovery pipeline.

## Figures and Tables

**Table 1:** Comparative Performance of Different Models.

Method	AUROC	AUPRC	Accuracy	Sensitivity	Specificity
BindingDB					
DeepConv-DTI	0.944 ± 0.004	0.925 ± 0.005	0.882 ± 0.007	0.873 ± 0.018	0.884 ± 0.009
GraphDTA	0.950 ± 0.003	0.934 ± 0.002	0.888 ± 0.005	0.882 ± 0.012	0.887 ± 0.008
MolTrans	0.952 ± 0.002	0.933 ± 0.004	0.887 ± 0.006	0.884 ± 0.019	0.883 ± 0.011
DrugBAN	0.956 ± 0.003	0.943 ± 0.003	0.897 ± 0.003	0.890 ± 0.015	0.896 ± 0.008
NFSA-DTI	0.951 ± 0.003	0.933 ± 0.004	0.883 ± 0.004	0.892 ± 0.008	0.908 ± 0.012
IHDFN-DTI	0.955 ± 0.002	0.939 ± 0.003	0.893 ± 0.003	0.884 ± 0.012	0.912 ± 0.009
LoF-DTI	**0.963 ± 0.005**	**0.947 ± 0.005**	**0.902 ± 0.002**	**0.896 ± 0.015**	**0.918 ± 0.007**
BioSNAP					
DeepConv-DTI	0.886 ± 0.006	0.890 ± 0.006	0.805 ± 0.009	0.760 ± 0.029	0.851 ± 0.011
GraphDTA	0.887 ± 0.008	0.890 ± 0.007	0.800 ± 0.007	0.745 ± 0.032	0.854 ± 0.025
MolTrans	0.890 ± 0.006	0.891 ± 0.005	0.804 ± 0.003	0.755 ± 0.021	0.846 ± 0.022
DrugBAN	0.903 ± 0.005	0.900 ± 0.004	0.836 ± 0.009	**0.825 ± 0.018**	0.849 ± 0.013
NFSA-DTI	0.897 ± 0.004	0.895 ± 0.008	0.832 ± 0.010	0.807 ± 0.015	0.844 ± 0.011
IHDFN-DTI	0.903 ± 0.005	**0.908 ± 0.006**	0.835 ± 0.007	0.815 ± 0.022	0.862 ± 0.008
LoF-DTI	**0.905 ± 0.003**	0.904 ± 0.002	**0.841 ± 0.005**	0.812 ± 0.020	**0.872 ± 0.014**
DAVIS					
DeepConvDTI	0.884 ± 0.008	0.299 ± 0.039	0.774 ± 0.012	0.754 ± 0.040	0.876 ± 0.013
DeepDTA	0.880 ± 0.007	0.301 ± 0.044	0.773 ± 0.010	0.765 ± 0.045	0.880 ± 0.024
MolTrans	0.892 ± 0.004	**0.371 ± 0.031**	0.779 ± 0.017	0.781 ± 0.023	0:878 ± 0.012
DrugBAN	0.892 ± 0.005	0.333 ± 0.039	0.770 ± 0.015	0.751 ± 0.024	0.869 ± 0.011
NFSA-DTI	0.884 ± 0.008	0.329 ± 0.028	0.774 ± 0.012	0.754 ± 0.030	0.866 ± 0.013
IHDFN-DTI	0.876 **±** 0.005	0.348 ± 0.032	0.778 ± 0.010	0.778 ± 0.013	0.874 ± 0.007
LoF-DTI	**0.894 ± 0.005**	0.354 ± 0.023	**0.782 ± 0.015**	**0.782 ± 0.015**	**0.882 ± 0.005**
Human					
DeepConvDTI	0.975 ± 0.002	0.969 ± 0.003	0.941 ± 0.002	0.915 ± 0.008	0.934 ± 0.015
DeepDTA	0.975 ± 0.002	0.969 ± 0.003	0.941 ± 0.002	0.915 ± 0.008	0.934 ± 0.015
MolTrans	0.973 ± 0.003	0.968 ± 0.003	0.943 ± 0.003	0.918 ± 0.007	0.936 ± 0.013
DrugBAN	0.981 ± 0.004	0.974 ± 0.006	0.938 ± 0.005	0.927 ± 0.011	0.938 ± 0.018
NFSA-DTI	0.980 ± 0.002	0.966 ± 0.005	0.943 ± 0.005	0.930 ± 0.007	0.947 ± 0.014
IHDFN-DTI	0.983 ± 0.004	**0.980 ± 0.003**	0.945 ± 0.002	0.938 ± 0.009	0.953 ± 0.006
LoF-DTI	**0.985 ± 0.004**	0.977 ± 0.002	**0.948 ± 0.007**	**0.944 ± 0.012**	**0.953 ± 0.008**

**Table 2:** Predicted Interactions for the Drug “Sertraline” and Protein “3QMN”.

Drug	Target	Prontein	Compound
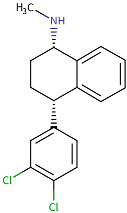	P31645 [37]	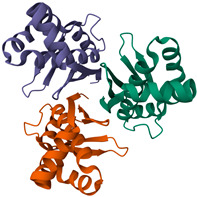	COA
	Q01950 [38]		A3P
	Q99720 [39]		MRD
	P23975 [40]		MPD
Sertralin	P08684 [41]	3QMN	ACT

**Table 3:** Key Hyperparameter Settings.

Hyperparameter	Setting
Optimizer	Adam
Learning rate	1 × 10^−5^
MAX_Epoch	100
BATCH_SIZE	64
Number of residual blocks	2
GIN layers	4
CNN kernel size	[3, 6, 9]
Heads of attention	4
Attention pooling size	3

**Table 4:** Datasets statistic.

Dataset	#Drugs	#Proteins	#Interactions
BindingDB	14,643	2623	49,200
BioSNAP	4510	2181	27,465
Human	2726	2001	6728
DAVIS	72	382	11,885

## Data Availability

The complete code of LoF-DTI and all associated datasets are available at: https://github.com/fbbgood/LoFDTI.git (accessed on 1 September 2025). The original contributions presented in this study are included in the article. Further inquiries can be directed to the corresponding authors.
